# Zolbetuximab-associated adverse event reporting signals in gastric cancer: a FAERS disproportionality analysis

**DOI:** 10.3389/fphar.2026.1887849

**Published:** 2026-09-10

**Authors:** Jingshun Zhang, Zhuo Zhen, Zhijun Li, Peng Zhu

**Affiliations:** Department of Gastrointestinal Surgery, The Second Affiliated Hospital of Chongqing Medical University, Chongqing, China

**Keywords:** CLDN18.2, FAERS, gastric cancer, PD-1 inhibitors, pharmacovigilance, zolbetuximab

## Abstract

**Background:**

Zolbetuximab has an established upper gastrointestinal tolerability profile, but the broader post-marketing spectrum of gastric mucosal events and findings related to albumin, protein loss, and fluid balance remains incompletely characterized.

**Methods:**

We conducted an indication-restricted, report-level US Food and Drug Administration Adverse Event Reporting System (FAERS) analysis from 2024 Q1 through 2026 Q1, comparing 597 zolbetuximab reports with 1,231 pooled nivolumab/pembrolizumab reports. Reporting odds ratios (RORs) were used for primary screening, with five contextual reporting backgrounds, adjusted Firth models, and chemotherapy-, drug-role-, geographic-, calendar-time-, and submission-channel analyses. Time to onset and co-reporting were summarized descriptively.

**Results:**

Among 1,041 screened preferred terms, 58 met the ROR criterion and 48 were clinically retained. The findings clustered into three groups: upper gastrointestinal and intake-related findings, gastric mucosal findings, and findings related to albumin, protein loss, and fluid balance. The eight *post hoc* focus preferred terms (PTs) retained support across all five backgrounds and the principal adjusted analyses. Gastritis was reported in 22 zolbetuximab *versus* 1 pooled PD-1 report (ROR 32.07, 95% CI 6.12–167.97), hypoalbuminemia in 72 *versus* 3 (ROR 48.43, 95% CI: 16.49–142.24), and protein-losing gastroenteropathy (PLE) in 10 *versus* 2 (ROR 8.79, 95% CI: 2.21–35.04). Japan accounted for 83.8% of zolbetuximab reports, with limited support outside Japan.

**Conclusion:**

The analysis reinforced established upper gastrointestinal findings. Gastritis and findings related to albumin, protein loss, and fluid balance require clinical confirmation, while protein-losing gastroenteropathy was reported sparsely. Overall, the results characterize reporting disproportionality in an early, Japan-dominant setting, not incidence or comparative clinical risk.

## 1 Introduction

Gastric cancer remains a substantial global health burden, with an estimated 968,350 new cases and 659,853 deaths in 2022 (Bray et al., 2024). For unresectable or metastatic adenocarcinoma, fluoropyrimidine–platinum (FP + Pt) chemotherapy remains a core systemic backbone, while treatment selection is increasingly biomarker-informed. HER2 status guides HER2-directed therapy, PD-L1 expression helps contextualize immune-checkpoint strategies, and claudin 18 isoform 2 (CLDN18.2) has emerged as an additional actionable target (Smyth et al., 2020; Ajani et al., 2025; Miyajima and Kawakami, 2025). Zolbetuximab, a CLDN18.2-directed monoclonal antibody, is approved with fluoropyrimidine- and platinum-containing chemotherapy for first-line treatment of adults with CLDN18.2-positive, HER2-negative, locally advanced unresectable or metastatic gastric or gastroesophageal-junction adenocarcinoma (U.S. Food and Drug Administration, 2025). Its introduction creates a need for early post-marketing evaluation as clinical exposure and spontaneous reporting practices evolve.

CLDN18.2 is a gastric-lineage tight-junction protein expressed in differentiated gastric mucosal epithelium. In normal tissue, its location within tight junctions limits zolbetuximab access to the target, whereas malignant transformation and loss of epithelial polarity can expose CLDN18.2 on the tumor-cell surface (Nakayama et al., 2024; Zeng et al., 2024). Zolbetuximab binds CLDN18.2 and promotes antibody-dependent cellular cytotoxicity and complement-dependent cytotoxicity. The randomized phase II FAST study and the multicohort phase II ILUSTRO study supported clinical development, followed by the phase III SPOTLIGHT and GLOW trials, which added zolbetuximab to mFOLFOX6 or CAPOX, respectively (Sahin et al., 2021; Klempner et al., 2023; Shitara et al., 2023; Shah et al., 2023). Across the pivotal program and current product information, nausea, vomiting, and decreased appetite were prominent tolerability findings. These well-characterized upper gastrointestinal events frame the remaining post-marketing questions.

Nausea and vomiting are well characterized, but the broader post-marketing spectrum remains less complete. Gastritis has been described in limited endoscopic and case-based evidence, while selected reports of protein-losing gastroenteropathy (PLE), alongside separate protein or albumin abnormalities, suggest additional clinically relevant phenomena that remain incompletely characterized (Yamamoto et al., 2025; Yanagimoto et al., 2025; Mitsui et al., 2025). This evidence is dispersed across studies with different designs and ascertainment methods and cannot define the complete post-marketing reporting spectrum. A systematic spontaneous-reporting analysis can map disproportionate reporting and organize these observations, although it cannot estimate incidence or comparative clinical risk or establish causality (Cutroneo et al., 2024; Fusaroli et al., 2024).

To address this gap, we conducted an indication-restricted US Food and Drug Administration Adverse Event Reporting System (FAERS) analysis to characterize zolbetuximab reporting disproportionality in gastric cancer and position the principal patterns alongside external clinical evidence. Pooled nivolumab and pembrolizumab reports served as the principal preferred term screening background to provide a relevant contemporary treatment context rather than a direct clinical comparison. Assessments by the National Institute for Health and Care Excellence and Canada's Drug Agency likewise considered PD-1 inhibitor-based chemotherapy in the contemporary treatment pathway (National Institute for Health and Care Excellence, 2025; Canada’s Drug Agency, 2025). Four additional contextual reporting backgrounds, together with adjusted and restricted analyses, were used to assess the dependence of findings on reporting context. FAERS date-field-derived time to onset and co-reporting were summarized descriptively.

## 2 Materials and methods

### 2.1 Data source and study design

We conducted a retrospective, report-level disproportionality study using individual FAERS case safety reports from 2024 Q1 through 2026 Q1. Quarterly ASCII files from demographic (DEMO), drug information (DRUG), indications (INDI), adverse reactions (REAC), patient outcomes (OUTC), report sources (RPSR), and therapy dates (THER) were analyzed together with the corresponding DELETE files. The quarterly extracts are raw, non-cumulative relational files, and source-file structure and field definitions were interpreted using the FDA quarterly data documentation (U.S. Food and Drug Administration, 2026). The analytical unit was one retained report. Each MedDRA preferred term (PT) was represented by its presence or absence at the report level, and repeated occurrences of the same PT within a report were counted once.

### 2.2 Report preprocessing and relational linkage

Reports associated with case identifiers (CASEIDs) in the FDA DELETE files were removed before selecting the case version. For each remaining CASEID, the latest version was selected deterministically using date received (FDA_DT), highest case version number (CASEVERSION), the numeric primary identifier (PRIMARYID), and source-quarter order, after which retained PRIMARYIDs were propagated to the related quarterly tables. DRUG rows were linked to INDI and THER rows within PRIMARYID by the corresponding drug sequence fields. The complete linkage rules and source fields are provided in Supplementary Table S1. Potential duplicates across different CASEIDs were examined conservatively using manufacturer identifiers and report signatures. Signature similarity alone was not used to remove a report.

### 2.3 Gastric cancer eligibility and cohort assignment

Disease eligibility was based on an author-defined normalized nine-term dictionary applied to INDI.INDI_PT. The qualifying indication had to be linked by PRIMARYID and drug sequence to the primary suspect (PS) row for the assigned study drug. Eligible concepts included gastric cancer and gastric adenocarcinoma, including stage, recurrent, metastatic, and HER2-positive gastric cancer terms. Gastroesophageal-junction or gastroesophageal indications, esophageal primary disease, metastasis to the stomach, gastric neuroendocrine or carcinoid disease, and conflicts with an additional non-gastric primary malignancy were outside the study scope. Exact included and out-of-scope normalized terms are listed in Supplementary Table S1.

Study drugs were identified from standardized drug-name information in DRUG using prespecified aliases, and their roles were obtained from DRUG.ROLE_COD. The principal index arm comprised reports with zolbetuximab coded as PS; the reference arm comprised reports with nivolumab and/or pembrolizumab coded as PS. A report containing both nivolumab and pembrolizumab contributed once to the pooled PD-1 reference arm. Reports with zolbetuximab and a pooled PD-1 study drug in PS or secondary suspect (SS) roles were excluded from the principal comparison. Study drugs recorded only as concomitant (C) or interacting (I) did not determine arm assignment. Chemotherapy, other targeted treatments, and supportive treatments were not exclusion criteria unless they created a cross-arm conflict regarding the assigned PS/SS designation of the study drug. Tislelizumab reports were analyzed separately in an exploratory comparison.

### 2.4 Reporting backgrounds and chemotherapy context

Pooled nivolumab/pembrolizumab reports formed the reference for the principal PT screen. Clinically retained PTs were subsequently presented in parallel across five reporting backgrounds: pooled PD-1, all gastric cancer reports without zolbetuximab in a valid role, non-PD-1 gastric cancer reports, FP + Pt-compatible reports, and chemotherapy-dominant reports. Each report contributed at most once within a background.

FP + Pt compatibility was assessed using drugs recorded in PS, SS, C, or I roles. Reports were classified as compatible when they contained both a fluoropyrimidine and oxaliplatin or cisplatin, or a recognized complete fluoropyrimidine–platinum (FP) regimen name; this represented report-level regimen compatibility rather than confirmed concurrent administration. Chemotherapy-dominant reports additionally excluded any prespecified immune-checkpoint inhibitor or targeted-treatment family recorded in these roles, while other co-reported treatments were permitted. The full fluoropyrimidine, platinum, regimen, immune-checkpoint, and targeted-treatment dictionaries are provided in Supplementary Table S1.

### 2.5 Adverse event mapping, PT screening, and clinical review

Reaction labels were obtained from REAC. PTs were harmonized to MedDRA version 29.0. Exact current PT matches were retained, and remaining exact authorized lower-level terms were assigned to their corresponding current PTs. Unmatched reaction strings were excluded from PT-level analyses without removing the associated reports or their other mapped reactions.

All PTs present in the principal comparison were screened, and every PT meeting the principal reporting odds ratio (ROR) criterion underwent clinical classification. One gastrointestinal surgeon performed the initial classification, and two additional gastrointestinal surgeons reviewed the classifications sequentially. Disagreements were resolved through discussion and consensus. Reviewers were not blinded to PT identity or the screening summaries. Because review was sequential rather than independent, inter-rater agreement could not be estimated. PTs were retained when they represented an adverse event or clinically relevant investigation finding; exclusions comprised disease-status or outcome terms, indication-related terms, and administration, dosing, or product-use terms.

**FIGURE 1:**
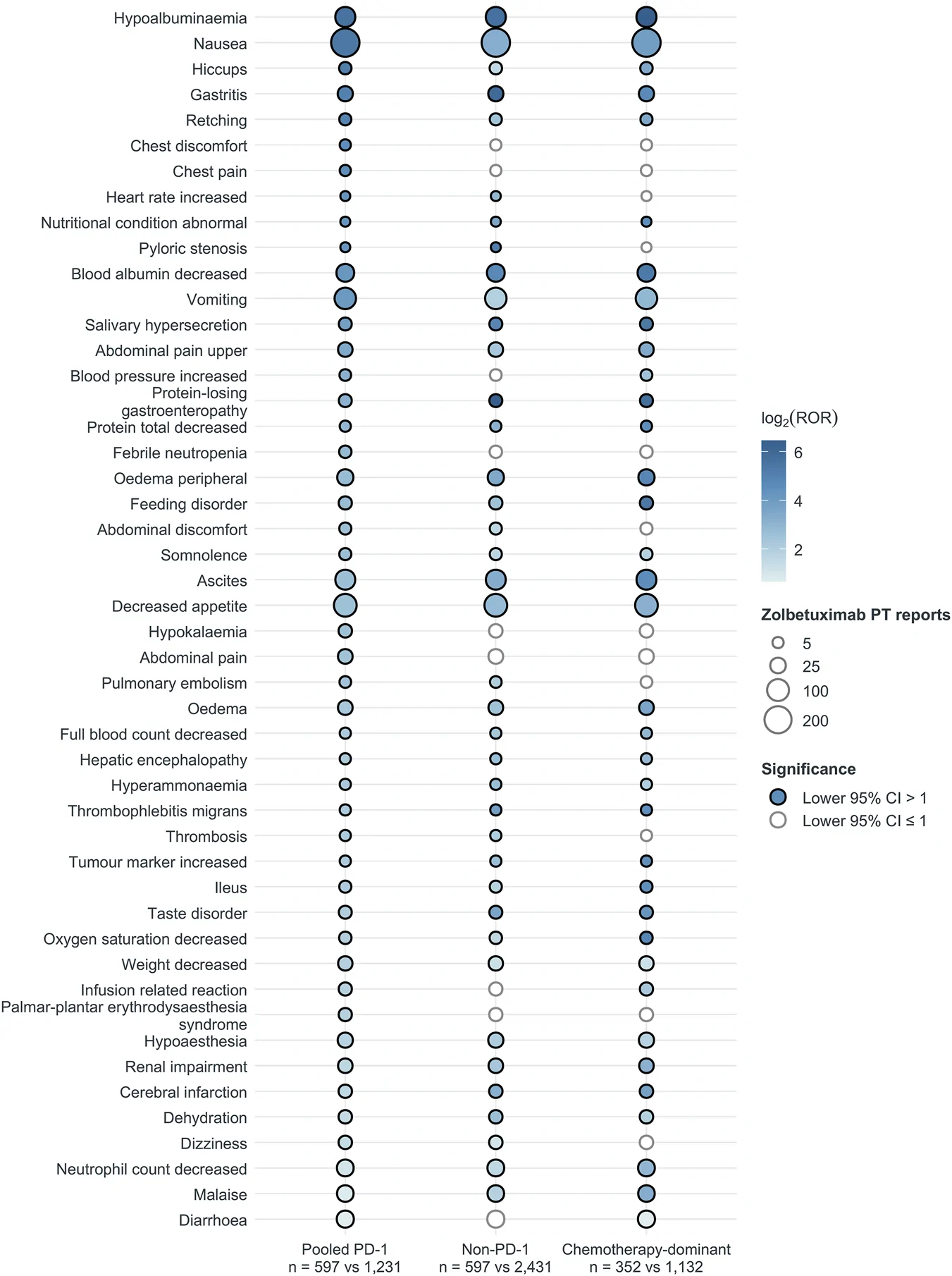
Unadjusted reporting odds ratios for the 48 clinically retained preferred terms across pooled PD-1, non-PD-1, and chemotherapy-dominant reporting backgrounds. Terms are ordered by descending pooled PD-1 ROR. The first and second denominators in each column header are the zolbetuximab and reference totals, respectively. Bubble area represents the number of zolbetuximab reports; colored bubbles indicate a lower 95% CI above 1, with fill intensity representing log_2_ (ROR), and hollow gray bubbles indicate a lower 95% CI of 1 or less. CI, confidence interval; PD-1, programmed cell death protein 1; PT, preferred term; ROR, reporting odds ratio.

A *post hoc* focus set of eight PTs was selected for concise main-text interpretation based on clinical relevance, representation of the principal reporting patterns, consistency across contextual and adjusted analyses, and external evidence positioning. All clinically retained PTs remained available in Figure 1 and Supplementary Table S2.

### 2.6 Disproportionality analysis

For each PT and reporting background, a report-level 2 × 2 table was constructed. The ROR was the principal screening metric; a PT met the screen when it occurred in at least three zolbetuximab reports, and the lower limit of its 95% confidence interval exceeded 1. A 0.5 continuity correction was applied to all four cells. The proportional reporting ratio (PRR) and information component (IC) were supportive metrics. PRR support required at least three zolbetuximab reports, PRR ≥2, and Pearson chi-square ≥4. IC was calculated as log_2_ [(a +0.5)/(E + 0.5)], where E = N_index (a + c)/(N_index + N_reference); IC support required at least three zolbetuximab reports and IC_025_ > 0. Two-sided Fisher exact *p*-values and Benjamini–Hochberg q-values were exploratory descriptors calculated across the complete screened PT universe in each background.

### 2.7 Adjusted and sensitivity analyses

For each clinically retained PT, Firth logistic regression (Firth, 1993) modeled report-level PT presence with study drug (zolbetuximab *versus* pooled PD-1) as the exposure. Covariates were age quintile, sex, reporting period, reporter region, and reporter category. Unknown levels were retained where applicable. The reporting period was derived from FAERS source-quarter metadata. Adjusted odds ratios (ORs) and profile-likelihood 95% confidence intervals were reported. Variable categories, reference levels, and model evaluability and convergence information are provided in Supplementary Table S3.

Three adjusted Firth sensitivities were performed: complete case analysis, restriction to FP + Pt-compatible reports, and inclusion of reports in which the assigned study drug was coded as PS or SS.

Restricted disproportionality analyses considered Japan, outside Japan, Japan reports submitted by healthcare professionals (Japan-HCP), and the common complete quarters from 2025 Q1 through 2026 Q1. Country was defined from OCCR_COUNTRY when available and REPORTER_COUNTRY otherwise. Japan-HCP was defined using reporter occupation codes detailed in Supplementary Table S3. The sole-PS analysis retained principal-cohort reports in which the assigned study drug was the only distinct drug concept coded PS; drugs in SS, C, or I roles did not affect this classification. Report-submission channel was derived from DEMO.REPT_COD, with expedited/periodic (EXP/PER) classified as manufacturer-submitted and DIR as direct-to-FDA. Restricted ROR, PRR, and IC estimates were calculated only when both study arms were evaluable.

Two exploratory Firth comparisons evaluated zolbetuximab against tislelizumab, first retaining missing reporter category as an explicit level and then omitting reporter category. A disease-definition sensitivity omitted the HER2-positive gastric cancer indication term while retaining all other cohort and analysis rules.

### 2.8 Time to onset, co-reporting, and external evidence assessment

Time to onset (TTO) was calculated as DEMO.EVENT_DT minus THER.START_DT for the assigned study drug. THER rows were linked by PRIMARYID and the study-drug sequence. Only complete dates were parsed; partial dates were not imputed, and the earliest complete eligible start date was used when more than one was available. Negative intervals were excluded from valid summaries. Zero-day intervals were retained as a separate descriptive category and excluded from positive-interval medians and interquartile ranges.

Within zolbetuximab reports, PT co-reporting was summarized at PRIMARYID level by shared-report counts, Jaccard indices, and overlap coefficients. Clinically prioritized PTs were contextualized using current regulatory labeling, the SPOTLIGHT and GLOW phase III trials, and selected primary post-marketing evidence to distinguish established, emerging, supportive but non-specific, and sparse hypothesis-generating findings.

### 2.9 Software

Analyses were performed in R version 4.5.3. Data processing and IC calculations used the data.table package version 1.18.4; Firth regression used logistf version 1.26.1; and figures were prepared with ggplot2 version 4.0.3 and patchwork version 1.3.2. Reaction terms were harmonized to MedDRA version 29.0.

## 3 Results

### 3.1 Cohort construction and PT classification

**FIGURE 2:**
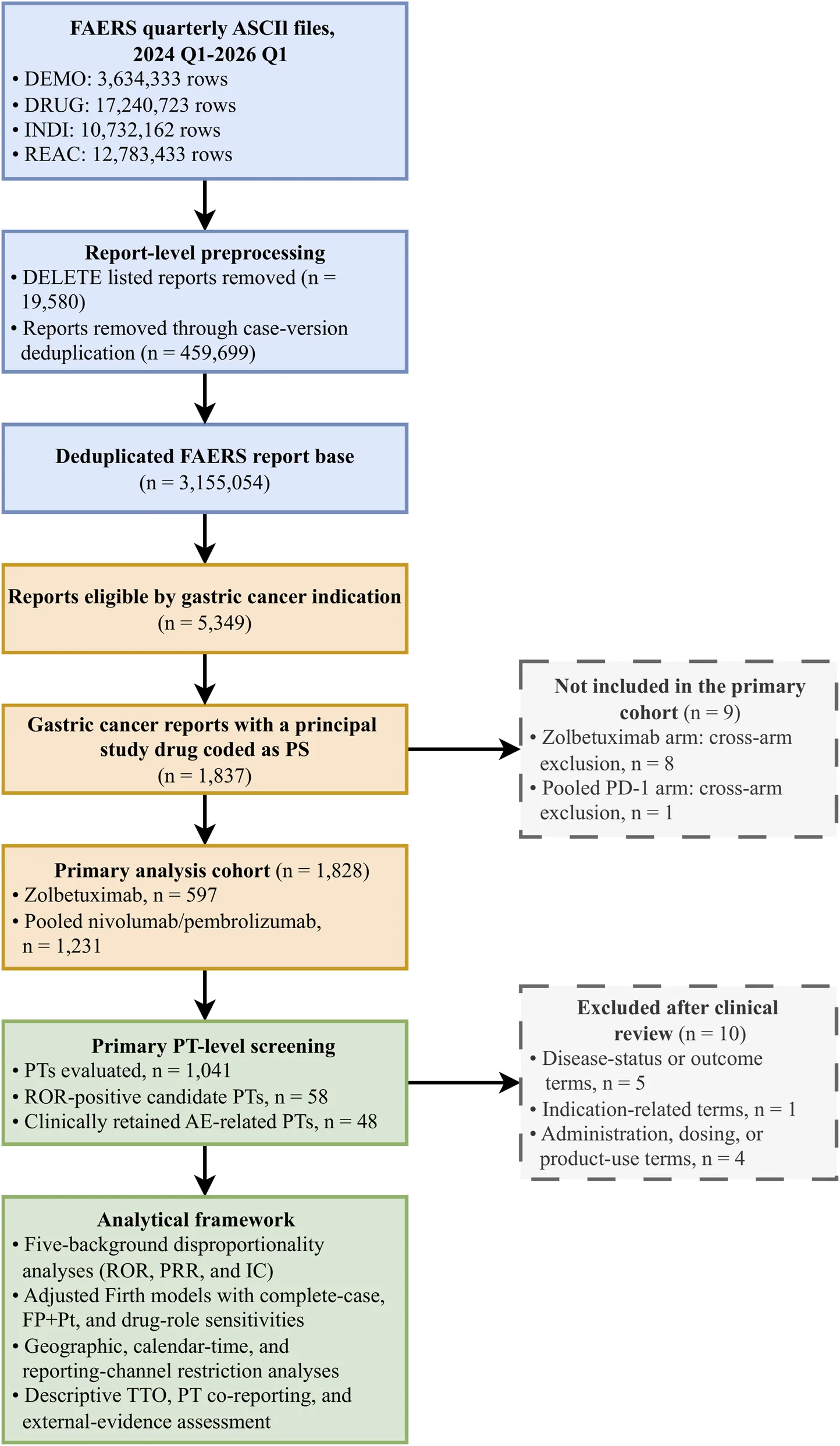
Cohort construction and analytical flow. FAERS, FDA Adverse Event Reporting System; PS, primary suspect; PD-1, programmed cell death protein 1; PT, preferred term; ROR, reporting odds ratio; PRR, proportional reporting ratio; IC, information component; FP + Pt, fluoropyrimidine plus platinum; TTO, time to onset. The diagram highlights DEMO, DRUG, INDI, and REAC as the core cohort construction files; OUTC, RPSR, and THER were also linked, and DELETE files were used during preprocessing. Detailed disease-scope exclusion flags were overlapping rather than sequential and are reported in Supplementary Table S1. Cross-arm exclusion indicates reports containing both zolbetuximab and a pooled PD-1 study drug in PS or SS roles.

The gastric cancer indication screen identified 5,349 eligible reports, of which 1,837 had a qualifying gastric cancer indication linked to a principal study drug coded PS. Cross-arm study-drug conflicts excluded eight reports from the zolbetuximab side and one from the pooled PD-1 side, leaving 597 zolbetuximab and 1,231 pooled nivolumab/pembrolizumab reports (1,828 reports overall). The conservative cross-CASEID audit identified no manufacturer-identifier-supported duplicate cluster, and no report was removed solely on the basis of a signature match (Figure 2; Supplementary Table S1).

The principal comparison contained 1,041 mapped PTs. Fifty-eight met the ROR screening criterion and underwent clinical classification; 48 were retained as AE-related PTs and 10 were excluded, comprising five disease-status or outcome terms, one indication-related term, and four administration, dosing, or product-use terms.

### 3.2 Reporting characteristics

**FIGURE 3:**
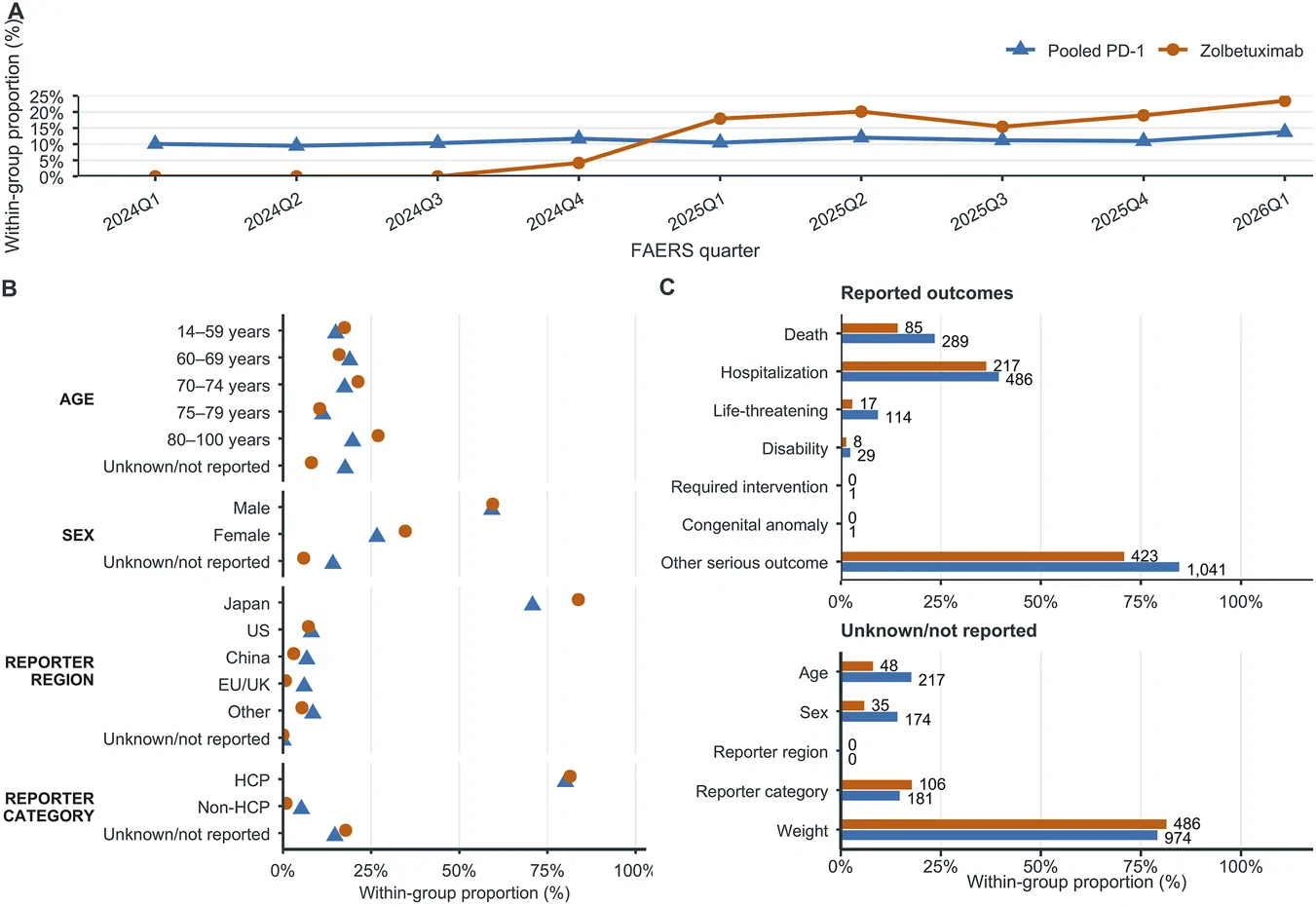
Reporting characteristics. **(A)** Within-group quarterly distribution of reports. **(B)** Within-group distributions of age group, sex, reporter region, and reporter category. **(C)** Within-group distributions of reported outcomes and missingness; outcome categories were not mutually exclusive. HCP, healthcare professional; PD-1, programmed cell death protein 1.

Zolbetuximab reports first appeared in 2024 Q4, and 572 of 597 (95.8%) occurred during the common 2025 Q1–2026 Q1 interval, compared with 719 of 1,231 (58.4%) pooled PD-1 reports. In 2026 Q1, 140 zolbetuximab reports (23.5%) and 169 pooled PD-1 reports (13.7%) were recorded. Among zolbetuximab reports, the largest age group was 80–100 years (161/597, 27.0%), followed by 70–74 years (127/597, 21.3%); age was unknown in 48/597 (8.0%), compared with 217/1,231 (17.6%) pooled PD-1 reports. Men accounted for 355/597 (59.5%) and 729/1,231 (59.2%) reports, respectively. Most reports originated from Japan (500/597, 83.8%; 871/1,231, 70.8%) and were submitted by HCPs (486/597, 81.4%; 986/1,231, 80.1%). The most frequently recorded outcomes were other serious outcome (423/597, 70.9%; 1,041/1,231, 84.6%), hospitalization (217/597, 36.3%; 486/1,231, 39.5%), and death (85/597, 14.2%; 289/1,231, 23.5%); outcome categories were not mutually exclusive. Weight was the most frequently missing characteristic (486/597, 81.4%; 974/1,231, 79.1%). These values describe FAERS reports rather than patient-level incidence or comparative clinical severity (Figure 3).

### 3.3 PT reporting patterns and focus PTs

**FIGURE 4:**
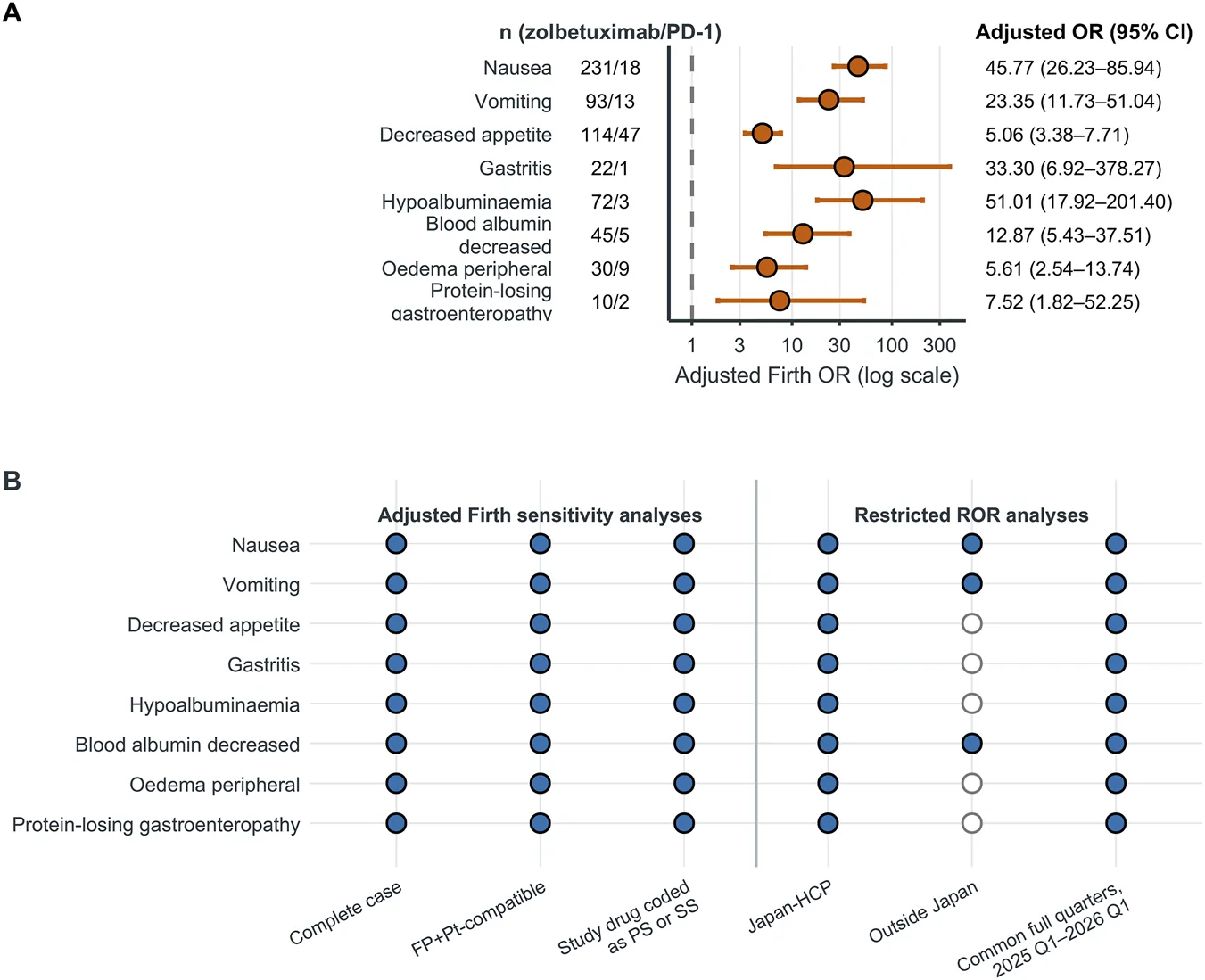
Adjusted and restricted analyses for the eight focus preferred terms. Panel A shows principal adjusted Firth odds ratios with profile-likelihood 95% CIs. Panel B shows complete case, FP + Pt-compatible, and PS-or-SS adjusted Firth sensitivities, followed by restricted RORs for Japan-HCP, outside Japan, and the complete 2025 Q1–2026 Q1 interval. Complete case required non-missing model covariates; Japan-HCP denotes Japan reports submitted by HCPs. Filled points indicate a lower 95% CI above 1; hollow points indicate that the 95% CI includes 1. CI, confidence interval; FP + Pt, fluoropyrimidine plus platinum; HCP, healthcare professional; OR, odds ratio; PS, primary suspect; PT, preferred term; ROR, reporting odds ratio; SS, secondary suspect.

Figure 1 presents unadjusted report-level RORs for all 48 retained PTs; adjusted Firth estimates are reported separately in Figure 4. Upper gastrointestinal and intake-related findings included nausea (231 zolbetuximab *versus* 18 pooled PD-1 reports; ROR: 41.43, 95% CI: 25.44–67.48), vomiting (93 *versus* 13; ROR: 16.73, 95% CI: 9.37–29.88), retching (7 *versus* 0; ROR: 31.28, 95% CI: 1.78–548.68), decreased appetite (114 *versus* 47; ROR: 5.91, 95% CI: 4.14–8.42), and feeding disorder. Gastric and abdominal findings included gastritis (22 *versus* 1; ROR: 32.07, 95% CI: 6.12–167.97), upper abdominal pain, and abdominal discomfort. Nutritional and hydration-related findings included nutritional condition abnormal, weight decreased, and dehydration. Albumin, protein-balance, and fluid-related findings included hypoalbuminemia (72 *versus* 3; ROR: 48.43, 95% CI: 16.49–142.24), blood albumin decreased (45 *versus* 5; ROR: 18.36, 95% CI: 7.54–44.74), protein-losing gastroenteropathy (10 *versus* 2; ROR: 8.79, 95% CI: 2.21–35.04), Oedema peripheral (30 *versus* 9; ROR: 6.92, 95% CI: 3.32–14.42), edema, and ascites.

Eight PTs were selected *post hoc* for focused main-text interpretation: nausea, vomiting, decreased appetite, gastritis, hypoalbuminemia, blood albumin decreased, Oedema peripheral, and protein-losing gastroenteropathy. Each met the ROR, PRR, and IC support criteria in all five reporting backgrounds (Table 1). All 48 clinically retained PTs are reported in Figure 1 and Supplementary Table S2.

**TABLE 1:** Reporting patterns for the eight focus preferred terms across five reporting backgrounds.

PT	Pooled PD-1	All non-zolbetuximab gastric cancer	Non-PD-1	FP + Pt-compatible	Chemotherapy-dominant
Nausea	231/597 vs. 18/1,231; ROR 41.43 (25.44–67.48); R+/P+/I+	231/597 vs. 241/4,695; ROR 11.65 (9.45–14.36); R+/P+/I+	231/597 vs. 151/2,431; ROR 9.51 (7.53–12.00); R+/P+/I+	165/364 vs. 135/2,441; ROR 14.12 (10.79–18.48); R+/P+/I+	163/352 vs. 61/1,132; ROR 15.03 (10.79–20.94); R+/P+/I+
Vomiting	93/597 vs. 13/1,231; ROR 16.73 (9.37–29.88); R+/P+/I+	93/597 vs. 172/4,695; ROR 4.86 (3.72–6.35); R+/P+/I+	93/597 vs. 110/2,431; ROR 3.89 (2.91–5.21); R+/P+/I+	68/364 vs. 96/2,441; ROR 5.62 (4.03–7.83); R+/P+/I+	66/352 vs. 34/1,132; ROR 7.39 (4.80–11.38); R+/P+/I+
Decreased appetite	114/597 vs. 47/1,231; ROR 5.91 (4.14–8.42); R+/P+/I+	114/597 vs. 172/4,695; ROR 6.21 (4.82–8.01); R+/P+/I+	114/597 vs. 78/2,431; ROR 7.10 (5.24–9.62); R+/P+/I+	95/364 vs. 103/2,441; ROR 8.01 (5.90–10.87); R+/P+/I+	92/352 vs. 42/1,132; ROR 9.11 (6.18–13.42); R+/P+/I+
Gastritis	22/597 vs. 1/1,231; ROR 32.07 (6.12–167.97); R+/P+/I+	22/597 vs. 2/4,695; ROR 73.40 (19.81–271.90); R+/P+/I+	22/597 vs. 1/2,431; ROR 63.35 (12.10–331.62); R+/P+/I+	11/364 vs. 2/2,441; ROR 31.74 (8.05–125.21); R+/P+/I+	11/352 vs. 1/1,132; ROR 25.40 (4.61–139.86); R+/P+/I+
Hypoalbuminemia	72/597 vs. 3/1,231; ROR 48.43 (16.49–142.24); R+/P+/I+	72/597 vs. 11/4,695; ROR 56.20 (29.97–105.38); R+/P+/I+	72/597 vs. 6/2,431; ROR 51.48 (22.95–115.50); R+/P+/I+	58/364 vs. 5/2,441; ROR 84.55 (34.99–204.29); R+/P+/I+	55/352 vs. 2/1,132; ROR 84.36 (23.60–301.49); R+/P+/I+
Blood albumin decreased	45/597 vs. 5/1,231; ROR 18.36 (7.54–44.74); R+/P+/I+	45/597 vs. 12/4,695; ROR 30.86 (16.40–58.06); R+/P+/I+	45/597 vs. 7/2,431; ROR 26.62 (12.23–57.96); R+/P+/I+	28/364 vs. 5/2,441; ROR 37.52 (14.95–94.14); R+/P+/I+	28/352 vs. 2/1,132; ROR 39.72 (10.84–145.54); R+/P+/I+
Oedema peripheral	30/597 vs. 9/1,231; ROR 6.92 (3.32–14.42); R+/P+/I+	30/597 vs. 32/4,695; ROR 7.71 (4.67–12.74); R+/P+/I+	30/597 vs. 11/2,431; ROR 11.31 (5.71–22.43); R+/P+/I+	20/364 vs. 21/2,441; ROR 6.70 (3.62–12.40); R+/P+/I+	20/352 vs. 2/1,132; ROR 27.88 (7.46–104.23); R+/P+/I+
Protein-losing gastroenteropathy	10/597 vs. 2/1,231; ROR 8.79 (2.21–35.04); R+/P+/I+	10/597 vs. 2/4,695; ROR 33.55 (8.42–133.64); R+/P+/I+	10/597 vs. 0/2,431; ROR 86.91 (5.09–1485.38); R+/P+/I+	8/364 vs. 2/2,441; ROR 23.27 (5.65–95.73); R+/P+/I+	8/352 vs. 0/1,132; ROR 55.89 (3.22–970.75); R+/P+/I+

Values are zolbetuximab n/N *versus* reference n/N, followed by ROR (95% CI). R+ indicates at least three zolbetuximab reports and a lower ROR (95% CI above 1); P+ indicates at least three zolbetuximab reports, PRR ≥2, and chi-square ≥4; I+ indicates at least three zolbetuximab reports and IC_025_ >0.

CI, confidence interval; IC, information component; PRR, proportional reporting ratio; PT, preferred term; ROR, reporting odds ratio.

### 3.4 Adjusted and sensitivity analyses

In contrast to the unadjusted RORs in Figure 1, Figure 4A shows covariate-adjusted Firth ORs. All eight focus PTs had 95% confidence intervals above 1 in the principal model. Representative estimates were 45.77 (95% CI: 26.23–85.94) for nausea, 23.35 (11.73–51.04) for vomiting, 5.06 (3.38–7.71) for decreased appetite, 33.30 (6.92–378.27) for gastritis, 51.01 (17.92–201.40) for hypoalbuminemia, and 7.52 (1.82–52.25) for protein-losing gastroenteropathy. Across all 48 retained PTs, 38 had a principal adjusted lower confidence limit above 1.

The focus findings were supported in the complete case, FP + Pt-compatible, and PS-or-SS Firth sensitivities. All 597 zolbetuximab reports and all 1,231 pooled PD-1 reports met the sole-PS definition; none contained another distinct PS drug concept. Consequently, the sole-PS ROR estimates reproduced the principal estimates, and all eight focus PTs retained support. The Japan-only and Japan-HCP restrictions supported all eight focus PTs; the Japan-HCP restriction included 425 zolbetuximab and 824 pooled PD-1 reports. All eight focus PTs also met the ROR criterion in the 2025 Q1–2026 Q1 common-quarter analysis, whereas three did so outside Japan. The manufacturer-submitted channel was evaluable for all eight focus PTs using 597 zolbetuximab and 1,226 pooled PD-1 reports, with all eight retaining ROR support. The direct-to-FDA channel included no zolbetuximab reports and five pooled PD-1 reports and was therefore not evaluable.

The exploratory tislelizumab comparisons included 597 zolbetuximab and 420 tislelizumab reports; sparse events produced imprecise estimates in both reporter category specifications. Omitting the HER2-positive gastric cancer indication term as an eligibility route left the zolbetuximab cohort at 597 reports and reduced the pooled PD-1 cohort from 1,231 to 1,205; all eight focus PTs retained support.

### 3.5 External evidence and clinical positioning

Table 2 summarizes the regulatory, phase III, and primary post-marketing evidence used to position the eight focus PTs by evidence tier.

**TABLE 2:** External evidence and clinical positioning of the eight *post hoc* focus preferred terms across current US FDA labeling, phase III trials, and primary post-marketing studies.

Preferred term	Evidence tier	Current US FDA label	Phase III context	Primary post-marketing evidence	Key caveat
Nausea	Established	Listed	Prominent with zolbetuximab	Management study (Narita et al., 2025)	Known event; chemotherapy background and stimulated reporting
Vomiting	Established	Listed	Prominent with zolbetuximab	Management study (Narita et al., 2025)	Known event; chemotherapy background and stimulated reporting
Decreased appetite	Established	Listed	More frequent with zolbetuximab	Observational and management studies (Yamamoto et al., 2025; Narita et al., 2025)	Non-specific in advanced gastric cancer and chemotherapy
Gastritis	Emerging	Not listed as a named reaction	Not prominent as a named trial event	Endoscopic cohort and case report (Yamamoto et al., 2025; Mitsui et al., 2025)	Predominantly Japanese observational evidence; limited support outside Japan
Hypoalbuminemia	Supportive but non-specific	Not listed as a named reaction; related decreased albumin laboratory abnormality is listed	Decreased albumin laboratory abnormality reported	Hypoalbuminemia cohort and albumin-kinetic cohort (Shimozaki et al., 2025; Ushimaru et al., 2026)	Multiple competing causes
Blood albumin decreased	Supportive but non-specific	Decreased albumin laboratory abnormality listed	Reported in laboratory safety data	Albumin-kinetic cohort (Ushimaru et al., 2026)	Overlaps clinically with hypoalbuminemia; not independent confirmation
Oedema peripheral	Supportive but non-specific	Reported	Reported; clinically non-specific	No distinct primary study identified	Multiple alternative causes of fluid imbalance
Protein-losing gastroenteropathy	Sparse, hypothesis-generating	Not listed	Not a named trial event	Case series and case report (Yanagimoto et al., 2025; Mitsui et al., 2025)	Very sparse evidence; case-level confirmation required

Evidence tiers were assigned *post hoc* for descriptive clinical interpretation and do not represent validated causal or confirmatory signal classifications.

### 3.6 Time to onset and co-reporting

Complete event and linked study-drug start dates were available for 408/597 zolbetuximab reports; 373 had nonnegative TTO, including 201 0-day and 172 positive intervals. Among focus PTs, positive-interval medians were 11 days for vomiting (IQR: 4–21; n = 13), 11.5 days for nausea (IQR: 2–21; n = 32), 13.5 days for decreased appetite (IQR: 4.5–22.5; n = 36), 14 days for Oedema peripheral (IQR: 7–49; n = 9), 20 days for blood albumin decreased (IQR: 14–68; n = 15), 21 days for hypoalbuminemia (IQR: 6–50; n = 19), and 30 days for gastritis (IQR: 7–55; n = 9). Protein-losing gastroenteropathy had a median of 68 days (IQR: 39–97), based on only two positive intervals.

Across all 1,128 unique unordered pairs among the 48 retained PTs, nausea and vomiting were co-reported in 72 reports (Jaccard 0.286; overlap coefficient 0.774), and nausea and decreased appetite were co-reported in 69 (0.250; 0.605). Hypoalbuminemia appeared with decreased appetite in 32 reports (0.208; 0.444), with Oedema peripheral in 16 (0.186; 0.533), and with protein-losing gastroenteropathy (PLE) in 6 (0.079; 0.600). The relatively high overlap coefficient involving PLE should be interpreted cautiously because PLE was reported infrequently. These metrics describe shared reporting and do not establish temporal sequence.

## 4 Discussion

This study organized zolbetuximab reporting in gastric cancer into three broad patterns: upper gastrointestinal and intake-related findings, gastric mucosal findings, and findings related to albumin, protein loss, and fluid balance. Nausea, vomiting, and decreased appetite were positioned as established because they are supported by pivotal trials, current product information, and management literature. Gastritis was positioned as emerging on the basis of informative but still limited post-marketing endoscopic evidence. Hypoalbuminemia, blood albumin decreased, and Oedema peripheral were considered supportive but non-specific because several competing explanations are common in advanced gastric cancer. Protein-losing gastroenteropathy (PLE) remained sparse and hypothesis-generating, with evidence derived mainly from selected cases (Shitara et al., 2023; Shah et al., 2023; Yamamoto et al., 2025; Ushimaru et al., 2026; Yanagimoto et al., 2025; Mitsui et al., 2025). Table 2 integrates these reporting patterns with regulatory, phase III, and primary post-marketing evidence.

The upper gastrointestinal findings were consistent with the established tolerability profile of zolbetuximab. Nausea, vomiting, and decreased appetite remained supported across contextual reporting backgrounds and adjusted analyses, in keeping with SPOTLIGHT, GLOW, and the current FDA label (Shitara et al., 2023; Shah et al., 2023; U.S. Food and Drug Administration, 2025). Clinical guidance and early practice reports emphasize anticipatory antiemetic prophylaxis around initial infusions, infusion-rate adjustment, prompt symptom reassessment, hydration, and support of oral intake so that chemotherapy and zolbetuximab can continue when clinically appropriate (Klempner et al., 2025; Narita et al., 2025; Shitara et al., 2026). Future cohorts should evaluate whether gastrectomy and residual-stomach status modify clinical presentation or management. Because 0-day records and incomplete dates were common, the timing summaries were interpreted descriptively.

Gastritis was reported in 22 zolbetuximab reports *versus* 1 pooled PD-1 report, with support across all 5 reporting backgrounds and adjusted analyses but limited support outside Japan. This finding adds a gastric mucosal component distinct from the established nausea and vomiting profile, although sparse reference events and the Japan-dominant reporting environment constrain interpretation. Because upper gastrointestinal symptoms alone cannot establish gastritis, direct mucosal assessment is important for distinguishing this finding from the established emesis profile. A Japanese cohort documented endoscopically confirmed inflammatory and erosive gastric changes during zolbetuximab-containing treatment (Yamamoto et al., 2025). Preclinical work supports the biological plausibility of gastric injury but cannot establish its human frequency or severity (Kinugasa et al., 2024). A reported case in which gastritis co-occurred with PLE suggests that gastric mucosal and protein loss findings may overlap in selected patients, without establishing a common sequence or mechanism (Mitsui et al., 2025). Prospective studies incorporating baseline and symptom-triggered endoscopy are needed to define the clinical spectrum and evaluate gastric-protective management strategies in routine care.

Findings related to albumin, protein loss, and fluid balance formed a recurring but clinically non-specific reporting pattern, while PLE may explain only a small subset of clinically confirmed cases. Hypoalbuminemia was reported in 72 zolbetuximab reports *versus* 3 pooled PD-1 reports and PLE in 10 *versus* 2; blood albumin decreased and edema peripheral also remained supported. Hypoalbuminemia and blood albumin decreased are clinically related coding concepts and therefore do not provide independent confirmation. Current product information identifies decreased albumin and peripheral edema as relevant findings, whereas PLE is not a named reaction (U.S. Food and Drug Administration, 2025). In a single-center retrospective exploratory cohort, Ushimaru et al. (2026) observed that serum albumin declined early during zolbetuximab-based treatment, without establishing PLE. A single-center cohort documented an early albumin decline and identified absence of prior total gastrectomy, capecitabine and oxaliplatin (CAPOX), higher body mass index, and nausea or vomiting during zolbetuximab introduction as factors associated with severe hypoalbuminemia, without establishing PLE (Shimozaki et al., 2025). Yanagimoto et al. (2025) reported two clinically documented cases of PLE during zolbetuximab treatment, providing evidence that PLE may occur in selected patients but not explaining the broader albumin- and fluid-related reporting pattern. PLE should therefore be considered only when supported by objective clinical evidence and should not be inferred from hypoalbuminemia or edema alone. Hypoalbuminemia and Oedema peripheral were shared in 16 reports (Jaccard 0.186); this metric indicates overlap within reports but establishes neither chronology nor a common mechanism. In advanced gastric cancer, reduced intake, vomiting, malnutrition, and cachexia complicate interpretation of albumin-related findings (Rosania et al., 2016). Tumor burden, organ dysfunction, and concomitant treatment may provide additional competing explanations. Prospective assessment should include serial albumin, body weight, dietary intake, nutritional evaluation, edema assessment, and objective testing for PLE to improve clinical attribution.

The five backgrounds: pooled PD-1, all non-zolbetuximab gastric cancer, non-PD-1, FP + Pt-compatible, and chemotherapy-dominant, showed how reporting context influenced effect magnitude. Nausea attenuated from an ROR of 41.43 against pooled PD-1 reports to approximately 9.5–15.0 across the other backgrounds, while vomiting showed a similar qualitative pattern. These analyses provided complementary reporting contexts. The FP + Pt-compatible and chemotherapy-dominant results, together with the complete case, PS/SS, sole-PS, and HER2-positive indication term sensitivities, did not materially alter the findings for the eight focus PTs. In contrast, interpretation of several non-focus PTs was more susceptible to disease- or treatment-related confounding. Ascites, diarrhea, neutrophil count decreased, malaise, and hypoesthesia were therefore not prioritized for focused interpretation; fluoropyrimidine–platinum exposure was a particular competing explanation for cytopenic, neurologic, and gastrointestinal findings (Cunningham et al., 2008; Yamada et al., 2015).

Applicability is shaped by geography, launch timing, and reporting route. Geographically, Japan accounted for 83.8% of zolbetuximab reports, and only three of eight focus PTs met the ROR criterion outside Japan. Temporally, 95.8% of zolbetuximab reports occurred during 2025 Q1–2026 Q1, although the focus findings remained supported when both arms were restricted to those quarters. Reporting patterns may evolve as exposure, clinical familiarity, publicity, and reporting practices change after launch. Oncology drugs have not shown a uniform classical Weber pattern, and the present concentration should therefore not be labeled a Weber effect (Arora et al., 2017). By reporting route, the manufacturer-submitted analysis supported the focus findings, whereas the direct-to-FDA channel was not evaluable because it contained no zolbetuximab reports. Taken together, these results primarily describe an early, predominantly Japanese post-marketing environment rather than a globally generalizable clinical safety profile.

Strengths of this study include the indication-restricted cohort, exact drug–indication linkage, and the use of multiple reporting backgrounds, adjusted models, and restricted analyses to examine the same reporting patterns from complementary perspectives. However, FAERS has no exposure denominator and is subject to underreporting, selective or stimulated reporting, potential duplicate reports, and variable data quality; it cannot support incidence, comparative risk, or causal estimates. Clinical detail was incomplete for biomarker intensity, treatment line, gastrectomy and residual-stomach status, baseline nutrition and albumin, tumor burden, regimen details, dose modification, and endoscopic confirmation, which constrained case-level attribution. The concentration of zolbetuximab reports in Japan and the early post-launch period limits generalizability, while overlapping reporting backgrounds and sparse cells make some estimates context-dependent or imprecise. Finally, the focus set was selected *post hoc*, and clinical classification used non-blinded sequential review rather than independent parallel ratings, so inter-rater agreement was not estimable. Ambiguous or incomplete dates limit interpretation of TTO, and co-reporting cannot establish temporal order, mechanism, or a common clinical syndrome. These limitations are intrinsic to spontaneous-report analysis and require the findings to be interpreted as reporting disproportionality rather than estimates of frequency or treatment effect.

## 5 Conclusion

In this indication-restricted FAERS analysis, zolbetuximab reports showed disproportionate reporting of established upper gastrointestinal events, gastric mucosal findings, and findings related to albumin, protein loss, and fluid balance across multiple reporting backgrounds. Gastritis was the clearest emerging finding; albumin-related events require careful clinical attribution, and protein-losing gastroenteropathy was reported sparsely. Because early post-marketing reports from Japan dominated the data, broader validation is needed, particularly with endoscopic assessment and serial evaluation of albumin, nutrition, and fluid status.

## Data Availability

Publicly available datasets were analyzed in this study. These data can be found at: Raw FAERS quarterly data: https://fis.fda.gov/extensions/FPD-QDE-FAERS/FPD-QDE-FAERS.html. Reproducibility code, dictionaries, and aggregate outputs: https://github.com/Sjzz114514/zolbetuximab-faers-reproducibility. Archived release v1.1.0: https://zenodo.org/records/21548960. DOI: 10.5281/zenodo.21548960.

## References

[B1] Ajani J. A. D’Amico T. A. Bentrem D. J. Corvera C. U. Das P. Enzinger P. C. (2025). Gastric cancer, version 2.2025, NCCN clinical practice guidelines in oncology. J. Natl. Compr. Canc. Netw. 23, 169–191. 10.6004/jnccn.2025.0022 40341199

[B2] Arora A. Jalali R. K. Vohora D. (2017). Relevance of the Weber effect in contemporary pharmacovigilance of oncology drugs. Ther. Clin. Risk Manag. 13, 1195–1203. 10.2147/TCRM.S137144 28979130 PMC5602442

[B3] Bray F. Laversanne M. Sung H. Ferlay J. Siegel R. L. Soerjomataram I. (2024). Global cancer statistics 2022: GLOBOCAN estimates of incidence and mortality worldwide for 36 cancers in 185 countries. CA Cancer J. Clin. 74, 229–263. 10.3322/caac.21834 38572751

[B4] Canada's Drug Agency (2025). Zolbetuximab for injection (Vyloy): reimbursement recommendation. Can. J. Health Technol. 5 (2), PC0338-000. Available online at: https://www.cda-amc.ca/zolbetuximab (Accessed July 25, 2026).

[B5] Cunningham D. Starling N. Rao S. Iveson T. Nicolson M. Coxon F. (2008). Capecitabine and oxaliplatin for advanced esophagogastric cancer. N. Engl. J. Med. 358, 36–46. 10.1056/NEJMoa073149 18172173

[B6] Cutroneo P. M. Sartori D. Tuccori M. Crisafulli S. Battini V. Carnovale C. (2024). Conducting and interpreting disproportionality analyses derived from spontaneous reporting systems. Front. Drug Saf. Regul. 3, 1323057. 10.3389/fdsfr.2023.1323057 40980108 PMC12443087

[B7] Firth D. (1993). Bias reduction of maximum likelihood estimates. Biometrika 80, 27–38. 10.1093/biomet/80.1.27

[B8] Fusaroli M. Salvo F. Begaud B. AlShammari T. M. Bate A. Battini V. (2024). The reporting of a disproportionality analysis for drug safety signal detection using individual case safety reports in pharmacovigilance (READUS-PV): explanation and elaboration. Drug Saf. 47, 585–599. 10.1007/s40264-024-01423-7 38713347 PMC11116264

[B9] Kinugasa F. Kajikawa S. Weng J. Ugawa T. Fushiki H. Yamanaka Y. (2024). Effect of antiemetics on zolbetuximab-induced gastric injury and emesis in ferrets. J. Pharmacol. Sci. 156, 161–170. 10.1016/j.jphs.2024.08.005 39313274

[B10] Klempner S. J. Lee K.-W. Shitara K. Metges J.-P. Lonardi S. Ilson D. H. (2023). ILUSTRO: phase II multicohort trial of zolbetuximab in patients with advanced or metastatic claudin 18.2-positive gastric or gastroesophageal junction adenocarcinoma. Clin. Cancer Res. 29, 3882–3891. 10.1158/1078-0432.CCR-23-0204 37490286 PMC10543966

[B11] Klempner S. J. Pazo-Cid R. A. Lonardi S. Swanson L. Arango M. J. Enzinger P. (2025). Consensus guidance for prevention and management of nausea and vomiting in patients treated with zolbetuximab plus chemotherapy: a RAND/UCLA modified Delphi panel study. ESMO Gastrointest. Oncol. 7, 100131. 10.1016/j.esmogo.2024.100131 41646481 PMC12836490

[B12] Mitsui Y. Sato Y. Shinomiya R. Sumida S. Fujimoto S. Okada A. (2025). Case report: zolbetuximab-induced gastritis with protein-losing gastroenteropathy and hypogammaglobulinemia: a case implicating IgA vasculitis. Front. Oncol. 15, 1644263. 10.3389/fonc.2025.1644263 40792275 PMC12336489

[B13] Miyajima Y. Kawakami T. (2025). Treatment selection for patients with HER2-negative metastatic gastric cancer expressing claudin 18.2 and PD-L1. Cancers (Basel) 17, 1120. 10.3390/cancers17071120 40227631 PMC11987827

[B14] Nakayama I. Qi C. Chen Y. Nakamura Y. Shen L. Shitara K. (2024). Claudin 18.2 as a novel therapeutic target. Nat. Rev. Clin. Oncol. 21, 354–369. 10.1038/s41571-024-00874-2 38503878

[B15] Narita Y. Mizuno T. Suda T. Kurono J. Ishizuka Y. Iida Y. (2025). Practical management of zolbetuximab administration: the Project VYLOY initiative. Cancers (Basel) 17, 1996. 10.3390/cancers17121996 40563646 PMC12190778

[B16] National Institute for Health and Care Excellence (2025). Zolbetuximab with Chemotherapy for Untreated Claudin-18.2-Positive HER2-Negative Unresectable Advanced Gastric or Gastro-Oesophageal Junction Adenocarcinoma. Technology Appraisal Guidance TA1046. Available online at: https://www.nice.org.uk/guidance/ta1046 (Accessed July 25, 2026). 40587646

[B17] Rosania R. Chiapponi C. Malfertheiner P. Venerito M. (2016). Nutrition in patients with gastric cancer: an update. Gastrointest. Tumors 2, 178–187. 10.1159/000445188 27403412 PMC4924460

[B18] Sahin U. Türeci Ö. Manikhas G. Lordick F. Rusyn A. Vynnychenko I. (2021). FAST: a randomised phase II study of zolbetuximab (IMAB362) plus EOX *versus* EOX alone for first-line treatment of advanced CLDN18.2-positive gastric and gastro-oesophageal adenocarcinoma. Ann. Oncol. 32, 609–619. 10.1016/j.annonc.2021.02.005 33610734

[B19] Shah M. A. Shitara K. Ajani J. A. Bang Y.-J. Enzinger P. Ilson D. (2023). Zolbetuximab plus CAPOX in CLDN18.2-positive gastric or gastroesophageal junction adenocarcinoma: the randomized, phase 3 GLOW trial. Nat. Med. 29, 2133–2141. 10.1038/s41591-023-02465-7 37524953 PMC10427418

[B20] Shimozaki K. Ooki A. Fukuoka S. Hirasawa T. Takamatsu M. Kawachi H. (2025). Hypoalbuminemia during zolbetuximab plus chemotherapy for claudin 18.2-positive advanced gastric cancer: a need for caution? ESMO gastrointest. Oncol. 10, 100257. 10.1016/j.esmogo.2025.100257

[B21] Shitara K. Lordick F. Bang Y.-J. Enzinger P. Ilson D. Shah M. A. (2023). Zolbetuximab plus mFOLFOX6 in patients with CLDN18.2-positive, HER2-negative, untreated, locally advanced unresectable or metastatic gastric or gastro-oesophageal junction adenocarcinoma (SPOTLIGHT): a multicentre, randomised, double-blind, phase 3 trial. Lancet 401, 1655–1668. 10.1016/S0140-6736(23)00620-7 37068504

[B22] Shitara K. Smyth E. Lordick F. Pophale R. Matney C. Matsangou M. (2026). Impact and effective management of nausea/vomiting on patients treated with zolbetuximab plus chemotherapy: insights from the phase III SPOTLIGHT and GLOW studies. ESMO Open 11, 105931. 10.1016/j.esmoop.2025.105931 41741112 PMC12947639

[B23] Smyth E. C. Nilsson M. Grabsch H. I. van Grieken N. C. Lordick F. (2020). Gastric cancer. Lancet 396, 635–648. 10.1016/S0140-6736(20)31288-5 32861308

[B24] U.S. Food and Drug Administration (2025). VYLOY (Zolbetuximab-clzb) Full Prescribing Information. BLA 761365. Available online at: https://www.accessdata.fda.gov/drugsatfda_docs/label/2025/761365s003lbl.pdf (Accessed July 25, 2026).

[B25] U.S. Food and Drug Administration (2026). FDA Adverse Event Monitoring System (AEMS) latest quarterly data files [formerly FDA Adverse Event Reporting System (FAERS)]. Available online at: https://www.fda.gov/drugs/fda-adverse-event-monitoring-system-aems/fda-adverse-event-monitoring-system-aems-latest-quarterly-data-files (Accessed July 25, 2026).

[B26] Ushimaru Y. Yamamoto K. Kudo T. Sakai D. Yanagimoto Y. Masuike Y. (2026). Zolbetuximab-based chemotherapy in CLDN18.2-positive gastric cancer: real-world tumor response and early albumin kinetics. Jpn. J. Clin. Oncol. 56, 695–703. 10.1093/jjco/hyag024 41686471

[B27] Yamada Y. Higuchi K. Nishikawa K. Gotoh M. Fuse N. Sugimoto N. (2015). Phase III study comparing oxaliplatin plus S-1 with cisplatin plus S-1 in chemotherapy-naïve patients with advanced gastric cancer. Ann. Oncol. 26, 141–148. 10.1093/annonc/mdu472 25316259

[B28] Yamamoto K. Owaki Y. Nakayama I. Sakamoto N. Ishizaka M. Kadota T. (2025). Characterization of early-onset gastritis during zolbetuximab-containing chemotherapy in CLDN18.2-positive gastric cancer. ESMO Open 10, 105805. 10.1016/j.esmoop.2025.105805 41033281 PMC12514510

[B29] Yanagimoto Y. Yamamoto K. Hara K. Masuike Y. Ushimaru Y. Kitamura M. (2025). Two cases of protein-losing enteropathy induced by zolbetuximab in patients with unresectable advanced gastric cancer. Jpn. J. Clin. Oncol. 55, 825–831. 10.1093/jjco/hyaf055 40194315

[B30] Zeng Y. Lockhart A. C. Jin R. U. (2024). The preclinical discovery and development of zolbetuximab for the treatment of gastric cancer. Expert Opin. Drug Discov. 19, 873–886. 10.1080/17460441.2024.2370332 38919123 PMC11938084

